# Educational outreach to general practitioners reduces children's asthma symptoms: a cluster randomised controlled trial

**DOI:** 10.1186/1748-5908-2-30

**Published:** 2007-09-24

**Authors:** Merrick Zwarenstein, Angeni Bheekie, Carl Lombard, George Swingler, Rodney Ehrlich, Martin Eccles, Michael Sladden, Sandra Pather, Jeremy Grimshaw, Andrew D Oxman

**Affiliations:** 1 Keenan Research Center, Li Ka Shing Knowledge Institute, St Michaels Hospital, Toronto, Canada; 2 Biostatistics Unit, Institute for Biostatistics, Medical Research Council, Cape Town, South Africa; 3 Department of Paediatrics, University of Cape Town, Cape Town, South Africa; 4 School of Public Health and Family Medicine, University of Cape Town, Cape Town, South Africa; 5 Centre for Health Services Research, University of Newcastle upon Tyne, Newcastle Upon Tyne, UK; 6 Ottawa Health Research Institute, University of Ottawa, Ottowa, Canada; 7 Informed Choice Research Department, Norwegian Health Services Research Centre, Oslo, Norway; 8 Department of Health Policy, Management and Evaluation, Faculty of Medicine, University of Toronto, Toronto, Canada; 9 Department of Dermatology, Leicester Royal Infirmary, University Hospitals of Leicester NHS Trust, Leicester, UK; 10 School of Pharmacy, University of the Western Cape, Cape Town, South Africa

## Abstract

**Background:**

Childhood asthma is common in Cape Town, a province of South Africa, but is underdiagnosed by general practitioners. Medications are often prescribed inappropriately, and care is episodic. The objective of this study is to assess the impact of educational outreach to general practitioners on asthma symptoms of children in their practice.

**Methods:**

This is a cluster randomised trial with general practices as the unit of intervention, randomisation, and analysis. The setting is Mitchells Plain (population 300,000), a dormitory town near Cape Town. Solo general practitioners, without nurse support, operate from storefront practices. Caregiver-reported symptom data were collected for 318 eligible children (2 to 17 years) with moderate to severe asthma, who were attending general practitioners in Mitchells Plain. One year post-intervention follow-up data were collected for 271 (85%) of these children in all 43 practices.

Practices randomised to intervention (21) received two 30-minute educational outreach visits by a trained pharmacist who left materials describing key interventions to improve asthma care. Intervention and control practices received the national childhood asthma guideline. Asthma severity was measured in a parent-completed survey administered through schools using a symptom frequency and severity scale. We compared intervention and control group children on the change in score from pre-to one-year post-intervention.

**Results:**

Symptom scores declined an additional 0.84 points in the intervention vs. control group (on a nine-point scale. p = 0.03). For every 12 children with asthma exposed to a doctor allocated to the intervention, one extra child will have substantially reduced symptoms.

**Conclusion:**

Educational outreach was accepted by general practitioners and was effective. It could be applied to other health care quality problems in this setting.

## Background

Asthma is common among children in Cape Town, South Africa, and is of great concern to the relatively poor communities where rates are highest, and where understanding of the disease and treatment adherence are poor [1,2]. Although South African guidelines for childhood asthma have been in the public domain for a decade [3], like elsewhere in the world [4], asthma is underdiagnosed by primary care doctors, prescribing is often inappropriate, and care is provided episodically [5].

In Cape Town, tax-funded public health care provides for the uninsured through a network of ambulatory care centres where nurses triage and doctors diagnose, prescribe, or refer to specialist care at public hospitals [6]. This system is free to children under the age of 13. However, for reasons of convenience, confidence, and personalised care, many residents of Mitchells Plain, the suburb in which this study took place, both with and without insurance, seek private sector primary care for their children.

Mitchells Plain is a dormitory town 30 km from Cape Town with a population of 300,000 people. Racially classified in the apartheid era as 'coloured', the residents suffered severe discrimination, with resulting social problems including high unemployment, overcrowded accommodation, poverty, alcohol and drug abuse, and criminal and family violence.

**Figure 1:**
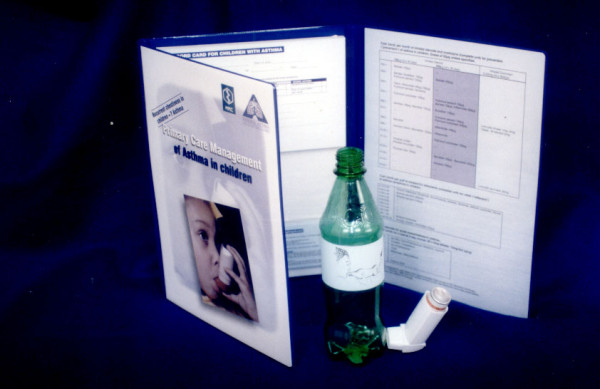
Support materials left behind for practitioner use.

Private healthcare in Mitchells Plain is usually provided by solo doctors without nurse support operating from storefront practices in the community (Figure 1). There is no formal registration list or roster system, and patients may move between several sources of primary care, including public sector clinics. Payment for private care provided to adults employed in the formal economy and their families is usually made by their employer-based health insurance, but for the informally employed and unemployed, payment is made by the patient in cash at the time of consultation. The cost of a single private sector primary care consultation, including medications, is about one day of average earnings for Mitchells Plain residents [7]. Consultations with local general practitioners and members of the South African National Asthma Education Programme, an organisation of asthma and allergy professionals, identified improvement in the quality of primary care as a priority for children with asthma in this setting.

Educational outreach (or academic detailing) [8] involves a trained messenger delivering one or more educational messages to a healthcare professional, and is a generally promising method of modifying health professional behaviour [9], though it has not been effective in changing the practise of primary care for childhood asthma in developed countries [10], and has never been evaluated in a lower- or middle-income setting for this purpose. This study evaluated the effect of academic detailing on the outcome of physician provided primary care for children with asthma in a cluster randomised controlled trial in an African setting.

## Methods

### Study design

The design was a cluster randomised controlled trial with the general practices as the unit of randomisation, intervention, and analysis. The study conforms to the Consort group recommendations for such trials [11] and was analysed on an intention to treat basis (see additional file 2). 

### Randomisation

A list of practices was composed in 1998, after identifying practitioners named in the baseline data from the medical register and telephone directory. Practices within the study area were numbered and randomised to two groups using a computer-generated list of random numbers.

### Inclusion criteria

We included all general practitioners, both practice principals and their hired doctors, working in private practice in Mitchells Plain. There were no multipartner practices. We included all schoolchildren up to age 17 living in the study area and their preschool siblings two years old or older with moderate to severe asthma. We determined their eligibility based on their answers to parent self-administered questionnaires and confirmatory face-to-face interviews conducted three months later.

### Intervention

The intervention was a tailored, multifaceted educational outreach intervention based on qualitative and survey research that identified barriers to the appropriate medical diagnosis and treatment of children with asthma in a similar nearby community (Table 1) [12]. It was aimed at improving the diagnosis, prescribing and follow-up care provided by private general practitioners to children with asthma.

**Table 1:** Barriers to diagnosis and treatment

**Physician reported barriers to diagnosis**	**Physician reported barriers to successful treatment**
Doctors find diagnostic criteria confusing	Fear of side effects of steroids
Insufficient consultation time for history, examination, peak flow measurement	Fear of addiction to inhalers
Organisation of care necessitates instant diagnosis (lack of continuity of care, leads to	Excessive antibiotic use
Organisation of care necessitates instant diagnosis (lack of continuity of care, leads to episodic approach cash payment and fee for service discourage repeat visits)	Cost of chronic medication
Masking by respiratory tract infection and by oral bronchodilator syrup	Poor patient understanding, adherence and inhalation technique
Stigmatised diagnosis	Passive smoke exposure in the home
High symptom tolerance in the community	Strong community belief in emotional cause of asthma discourages medical treatment
	Doctor hopping prevents follow-up

The intervention contained eight key messages to convey to practitioners. We included only messages related to clinical behaviours that we believed to be largely under the control of the practitioner; in other words, free of external constraints and thus amenable to change by the practitioner (Table 2).

**Table 2:** The eight key messages delivered to general practitioners

Rely on a history of recurrent chestiness as a diagnostic indicator
Preferentially prescribe inhaler over oral therapy
Prescribe using a treatment algorithm based on asthma severity
Appropriately prescribe inhaled anti-inflammatory therapy
Demonstrate and encourage patients to use home-made spacers
Prescribe short-course oral steroids for exacerbations of asthma
Recall patients for regular follow-up care
Encourage parents to avoid smoking near asthmatic children

**Figure 2:**
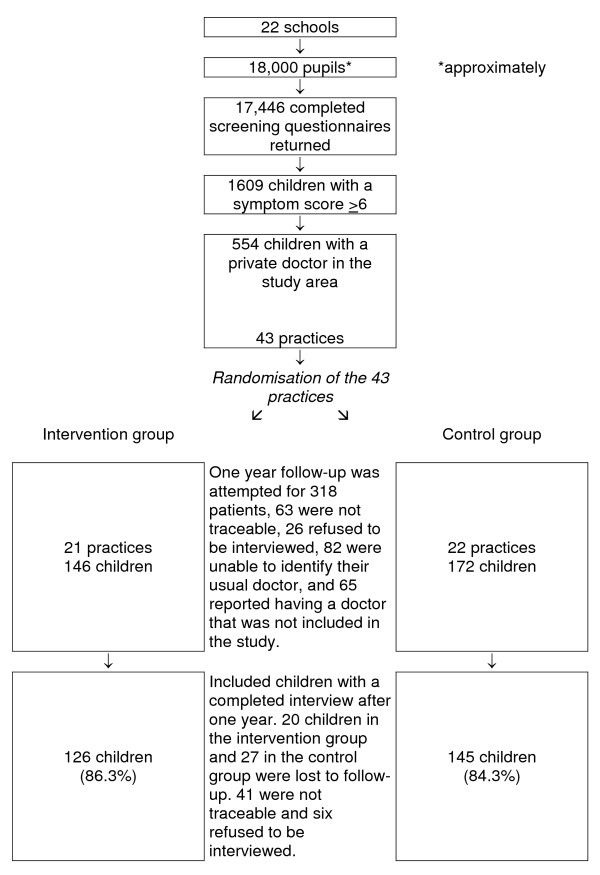
Trial flow diagram.

The intervention was delivered during 1998 to individual practitioners by a pharmacist trained in the methods of academic detailing. A first visit took 30 minutes with a repeat visit of similar duration conducted three months later. At the first visit, the pharmacist used a visual aid, a set of printed glossy materials similar to those used by pharmaceutical company representatives, structured as a plastic laminated desk blotter, on which the key messages were outlined (Figure 2). The blotter was left behind in the practice, along with instructions for modifying a 500 ml plastic soft drink bottle to attach to a pressurised metered dose inhaler as a volume increasing spacer and an actual example of one such spacer. (Spacers reduce the difficulties children have in coordinating their breathing with triggering of the inhaler).

### Outcome measurement

The principal outcome for the trial was the change in an individual child's asthma symptom score reported by the parent or guardian before and after the intervention. The South African Consensus guidelines use a severity grading of childhood asthma based on frequency of attacks of tight chest, nocturnal coughing, and nocturnal waking, hospital admissions, and peak flow rate. The three attack frequency questions were amenable for use in a brief interview, and were weighted by zero to three points, according to frequency of episodes in the previous 12 months, using weights developed in a previous study in this community [6], where one to two episodes equalled one point, three episodes equalled two points, and four or more episodes equalled three points.

The score obtained for these three frequency questions was added to give a total score. The maximum score attainable was nine points and the minimum score attainable was zero points. Children with the highest pre-intervention symptom scores were included until the desired sample size for the trial was obtained. In contrast with the specific symptom questions in the score, we also asked parents a number of questions designed to measure their perception of the child's asthma severity and the effect of asthma on participation in usual activities such as school (see additional file 1).

### Data collection

Initially, parents completed a self-administered asthma screening survey for their primary school children and younger siblings, distributed via the primary schools in the study area. In later rounds, parents of those children identified from the returned screening questionnaires as having moderate or severe asthma symptom scores and a regular private doctor were interviewed face-to-face in their homes by experienced and trained fieldworkers before the intervention and one year later (1999). Self administered questionnaires and interviews were completed in the respondents chosen language. Interviewers and parents were blinded as to the allocation of practitioners.

The process was designed to obtain a group of children whose parents consistently reported their usual source of care as one or more participating private practitioners, and whose pre-intervention symptoms were compatible with moderate to severe asthma. We thus excluded at screening children whose parents reported no usual private general practitioner, or whose home address was outside Mitchells Plain. At the baseline face-to-face interview, we also excluded children whose parents reported that their child's usual family practitioner was outside the study area, and at follow-up face-to-face interview, we further excluded children whose parents identified as their practitioner a doctor who was not on our list of randomised practitioners.

### Sample size

We wished to detect a clinically meaningful improvement, 0.5 standard deviations, in the symptom score between intervention and control groups [13]. We assumed a standard deviation of one, 5% significance, 80% power, and an intracluster correlation coefficient of 0.17 (similar to primary care practices in other countries [14]). With 43 available clusters, we needed 15 patients per cluster, for a total of 280 patients [15].

### Statistical analysis

Data were collected on paper and entered into a computer. They were managed and analysed using SAS (Version 8.2. SAS Institute Inc., Cary, NC, USA). We report univariate descriptions, cross-tabulations with chi-squares, and, for adjusted analysis of the principal outcome, the asthma symptom score, we report a linear regression analysis on the change in asthma symptom score from pre-to post-intervention evaluation. The clustered design was accommodated by fitting the generalised estimating equation version of the linear regression model with an exchangeable working correlation model. To check the findings of the linear model on the change scores, an ordinal logistic regression analysis using the proportional odds model was carried out on the post intervention score [16]. Since the results were corroborated, only the linear model with change scores is reported. The variables included in the model, decided *a priori*, were the baseline score and the number of visits to the usual practitioner during the study period. We conducted the adjusted analysis using Proc GENMOD in SAS.

### Ethics and consent

Permission to contact parents of schoolchildren to complete a survey was obtained from the Department of Education for the province, and then, during an information meeting at each of the primary schools in the study area, permission was sought from, and granted by, each school principal and class teacher.

Plain language explanations of the purpose of the study and its voluntary nature were included with the self-administered questionnaire sent to parents. Return of the questionnaire indicated consent to use the data. During home visits, the study was again explained and confirmatory verbal consent was obtained.

Intervention group practitioners were contacted to obtain an appointment for the academic detailing visit. At the beginning of the visit, some well-known problems of asthma care and the academic detailing intervention were outlined, and the doctor's participation in an evaluation of the intervention was invited. No monetary reward was offered. Control group practices received a hand-delivered copy of the then current South African childhood asthma guideline. Because practitioner records were not used for identification or follow-up of children with asthma, no permission was required from practitioners for this element of the study.

Ethical approval for the study was granted by the Medical Research Council of South Africa Ethics Committee.

## Results

We identified 43 practices at the start of the study, 21 of which were randomised to intervention, and 22 to the control group (comparability, Tables 3 and 4; and flow diagram, Figure 3). No practices were lost to follow-up. One intervention group practitioner refused to take part in the intervention, and one moved out of the area and was replaced in his practice by another doctor. The replacement practitioner was not offered academic detailing. The trial was analysed by intention to treat, including the patients of these practitioners.

**Table 3:** Comparability of practitioners and practices

	**Intervention**	**Control**	**p-value**
	(21 practices)	(22 practices)	

Up to 10 years since physician registered	9	1	0.1*
11–20 years since registration	11	9	
More than 20 years since registration	12	13	
Male physicians	24	21	0.17^†^
Female physicians	8	2	
0 – 5 children with asthma in practice	12	14	0.76^†^
>5 children with asthma in practice	9	8	

*Chi square = 5.3

^†^Fisher's exact test

**Table 4:** Comparability of children between study arms

	**Intervention**	**Control**	**p-value**
Mean age in years (range)	7.5 (1 – 17)	7.7 (1 – 17)	0.76*
Number of girls (%)	63 (50)	75 (48)	0.68^†^
Number of boys (%)	63 (50)	70 (52)	

*Generalised estimating equation analysis

† Fisher's exact test

Children in the intervention group (n = 126) ranged from 1 to 17 years of age (median, 7.5 years), with an equal number of boys and girls. Children in the control group (n = 145) also ranged from 1 to 17 years of age (median, 7.7 years), with 70 boys and 75 girls (Table 4).

### Symptom score

The principal outcome measure for the trial was the change in asthma symptom score over the one year between baseline and follow up surveys, during which period the intervention took place. There was substantial decline in reported symptoms over one year in the intervention group (4.08) and the control group (3.24) (Table 5). The decline in symptom score was 0.84 points greater in the intervention group than in the control (p = 0.03).

**Table 5:** Change in score

	**Intervention Mean(SE)**	**Control Mean(SE)**	**Estimated Intervention Effect Mean (95%CI)**	**p-value from GEE model**
Pre-intervention mean score	7.71 (0.11)	7.48 (0.09)		
Post-intervention mean score	3.63 (0.26)	4.24 (0.27)		
Pre-post difference	4.08 (0.23)	3.24 (0.30)	0.84 (0.10; 1.58)	0.03
Pre-post difference adjusted for number of physician visits	4.10 (0.18)	3.25(0.27)	0.85 (0.21 ;1.48)	0.01

### Adjusted analyses

At baseline, the mean asthma symptom score was higher in the intervention group, suggesting a slightly more severe distribution of disease in that group (Table 5). Adjusting for the baseline difference using ordinal logistic regression produces an odds ratio (rather than a mean difference) that is consistent with the unadjusted results (OR = 1.48, 95% CI 1.00 – 2.20, p = 0.049).

To investigate the effect on the principal outcome measure of the number of visits to the specified physician, this factor was added to the model as a linear effect. The estimated intervention effect (Table 5) was close to the unadjusted intention to treat analysis, but with a narrower confidence interval. Children with more frequent physician visits had a non-significant tendency towards smaller symptom changes (slope of -1.41, p = 0.10). The slope was similar in both intervention and control groups.

### Subjective assessments of well-being and impact

There were no significant differences between the intervention and control group respondents in their subjective assessment of their children's overall asthma severity in comparison with the previous year, nor in their ability to undertake normal school activities (Table 6).

**Table 6:** Subjective assessments of well-being and impact

	**Intervention % (n)**	**Control % (n)**	p-value*
Breathing problems improved compared to 12 months previously	74% (90)	73% (99)	0.849
Chest problems create little or no problem for child school activities	98% (119)	97% (130)	0.686

*Fisher's exact test

## Discussion

This study appears to be the first to show improvements in childhood asthma symptoms in a lower or middle income country following educational outreach. The measure of outcome has not been formally validated. However, it is a simple set of symptoms that are a commonly occurring feature of the disease, well known to parents, and thus the measure has high face validity. In addition, when used in a randomised trial as here, error and poor recall would bias towards a null effect. The substantial effect on symptoms which we found is therefore likely to be an underestimate of the true effect of this intervention.

The improvement in asthma symptoms is unlikely to be explained by bias, because the intervention and control patients and practitioners were comparable at outset, the analysis was by intention to treat, there was no loss to follow-up among practices, and little differential loss to follow-up of patients.

Identification of patients through a school-based survey rather than practice records protected the study against possible bias from differences in recordkeeping standards and reduced logistical difficulties. Collecting data from schools also minimised the impact of the trial on physicians' awareness and possible changes in behaviour due to that awareness. It also enabled us to measure the effect of the intervention on unselected practitioners rather than on volunteers. The results are therefore likely to be applicable and useful in similar settings.

Only one practitioner refused the outreach visits, and his patients were analysed in the allocated group, and the result is thus a real world finding. Academic detailing was a welcome intervention in practice settings such as these, as demonstrated in accompanying qualitative research reported elsewhere [12]. It is likely that this finding is applicable wherever physicians are relatively isolated from their colleagues.

The decline in reported symptoms over one year in both the intervention and control groups was likely due to aging of the children in the study, and may also be due to regression to the mean. The intervention reduced asthma symptoms even further in the intervention group (0.84). For a cut point of six or above on the nine point symptom scale that was used, one additional child in the intervention practices benefited for every 12 children cared for in those practices

In contrast with the improvement in asthma symptoms, there were no improvements in well-being or burden of the disease, as measured by global questions. This might be due to the high threshold of response to illness in a relatively poor community, the insensitivity of global questions in comparison to the very specific and memorable events tapped by the symptom severity questions in the scale, or the lack of power for the global questions, which were dichotomous.

Few other studies of educational outreach have measured health outcomes, few have been undertaken in private practice in a poor urban community, and none have measured outcomes using a school-based survey rather than medical records or administrative databases. These pragmatic characteristics of this trial increase its relevance in this setting, widen its applicability, and demonstrate that it is possible to conduct rigorous evaluations of behaviour change interventions in low and middle income settings.

Alongside this study we explored physicians' perceptions of the outreach visits through qualitative means, described in reports available elsewhere [12]. Although it would have been too complicating in this trial to have used survey instruments to study the processes leading to behaviour change, other researchers may consider incorporating such embedded evaluations of these processes in future studies.

The setting in which the intervention was applied was a difficult one with individual storefront practices, where physicians have very little organisational support. The main source of treatment information in such practices is likely to be from drug company representatives. Using the familiar drug company detailing model, we were able to meet with practitioners and tailor the message to their needs and conceptions of the problem.

## Conclusion

This intervention appeared affordable for a low-to middle-income country like South Africa, and would add about 0.01% to the annual public sector healthcare budget for each condition at which outreach was aimed, if used nationwide once per physician per annum. We also have successfully used an educational outreach approach to nurse clinicians in even more impoverished and rural parts of South Africa [17]. Policymakers in similar settings could consider introducing publicly funded outreach visits as a potentially cost-effective way to improve the quality of care given by isolated providers in both public and private health care sectors, and thereby, improve health outcomes.

## Competing interests

ME is co-editor in chief of Implementation Science, JG is a member of the editorial board. All editorial decisions on this paper were made independently by co-editor in chief, Brian Mittman, not an author. All other authors have nothing to declare.

## Authors' contributions

MZ conceived the project, led the design of the interventions, the trial, the barrier and pilot studies, contributed to analysis, and wrote the drafts. All other authors contributed to editing and approval of the final version, and in addition, AB contributed to design of the interventions and qualitative evaluations, conducted the intervention and led the fieldwork; CL led all statistical aspects of design and analysis, while GS, RE, AO, SP, MS, ME, and JG contributed to conception of the project and the intervention, supported design of the trial, and contributed to analysis.

## Supplementary Material

Additional file 2 CONSORT checklist. List of items to include when reporting a randomized trial. Click here for file

Additional file 1 Survey instrument. Questions on asthma symptomatology and demography of included children. Click here for file
