# Supplementary material for: Educational outreach to general practitioners reduces children's asthma symptoms: a cluster randomised controlled trial
Source: Implement Sci. 2007 Sep 24;2:30. doi: 10.1186/1748-5908-2-30 (PMC2200659; doi:10.1186/1748-5908-2-30)
Supplement: Additional file 1 — Survey instrument. Questions on asthma symptomatology and demography of included children. [file 1748-5908-2-30-S1.doc]

# APPENDIX 1

A. Self-Administered Questionnaire

1. First names of child

2. Surname of child

3. Sex of child

4. How old is the child today in years?

5. What is the child’s date of birth? Day Month Year

6. Address of child

7. You are the child’s ? Mother

Father

Grandmother

Other (specify)

8. In the last 12 months, has this child had tight chest or wheezing or whistling in the chest? Yes

No

9. How many times has this child had tight chest or wheezing or whistling in the chest in the last 12 months? None

1 or 2 times

3 times

4 or more times

10. In the last 12 months has this child had a troublesome dry cough at night, that was not from a cold or a chest infection? Yes

No

11. How many times in the last 12 months has this child had a troublesome dry cough at night that was not from a cold or chest infection? None

1 or 2 times

3 times

4 or more times

12. In the last 12 months has this child woken up at night due to a tight chest or wheezing or whistling in the chest? Yes

No

13. How many times has this child woken up due to tight chest or wheezing or whistling in the chest in the last 12 months? None

1 or 2 times

3 times

4 or more times

14. Do you have a usual private doctor for this child? Yes

No

15. How many times in the last 12 months has this child been to this usual doctor for chest or breathing problems? None

1 or 2 times

3 times

4 or more times

16. How many times in the last 12 months has the child been to any other doctor for chest and breathing problems? None

1 or 2 times

3 times

4 or more times

17. Has this child ever had asthma? Yes

No

18. How many babies and children younger than 18 years of age live in this house?

B. Interviewer-administered Questionnaire

All questions except those numbered 4 and 18 in the self-administered questionnaire were

repeated in the interviewer-administered questionnaire.
